# Neutrophil-to-Lymphocyte Ratio as a Potential Predictive Marker for Epileptic Seizures: Unveiling the “V”-Shaped Link

**DOI:** 10.1155/mi/2247724

**Published:** 2025-03-19

**Authors:** Xianmei Wang, Yang Zhang, Yi Ye, Long Wang, Yi Xu, Siying Ren, Likun Wang, Guofeng Wu

**Affiliations:** ^1^Basic Medical College, Guizhou Medical University, Guiyang, Guizhou, China; ^2^School of Nursing, Guizhou Medical University, Guiyang, Guizhou, China; ^3^School of Clinical Medicine, Guizhou Medical University, Guiyang, Guizhou, China; ^4^Department of Emergency, The Affiliated Hospital of Guizhou Medical University, Guiyang, Guizhou, China; ^5^Department of Emergency, The 300 Hospital of Guihang, Guiyang, Guizhou, China

**Keywords:** epilepsy, inflammation, neutrophil-to-lymphocyte ratio, seizure

## Abstract

**Objective:** The neutrophil-to-lymphocyte ratio (NLR) is an important marker of inflammation. An increased NLR has been detected in the blood of patients with epilepsy. However, the correlation between varying NLRs and epileptic seizures (ESs) is still unclear.

**Methods:** A retrospective analysis was conducted, and patients were divided into two groups based on whether they had ES upon admission. Comprehensive data were collected, including routine blood tests, demographic information, and medical histories. The NLR was calculated by dividing the percentage of neutrophils by the percentage of lymphocytes.

**Results:** In total, 414 patients were included (284 males, 151 females; aged 18–90 years), with 271 in the experimental group and 143 in the control group. No significant differences in the NLR were found between the groups (*p*=0.164). Nonetheless, when NLRs were categorized, a “V”-shaped link with ES was observed. An NLR of 2–3 correlated with the lowest seizure incidence. Higher NLRs were associated with increased neutrophil and decreased lymphocyte counts. Patients with an NLR < 2 had the lowest infection rates and the highest cerebrovascular disease exposure, whereas those with an NLR > 3 had the highest infection rates and were the oldest.

**Conclusions:** NLR modifications can serve as potential predictive markers for ES. However, the relationship between the NLR and ES is not linear. The factors contributing to these variations are multiple and complex. An NLR of 2–3 may represent an equilibrium point. An elevated NLR indicates pronounced inflammatory responses, while a low NLR can have more complex causes.

## 1. Introduction

Epilepsy is the most common chronic neurological disorder and involves recurrent unprovoked seizures [1]. Annually, ~49 per 100,000 individuals are diagnosed worldwide, with rates reaching 139 per 100,000 individuals in low- and middle-income countries [2]. It affects all ages but is most common in infants and adults aged >50 years [3]. Current treatments focus on controlling seizures, yet 30% of patients experience uncontrolled seizures [4, 5]. Epileptic seizures (ESs) can lead to serious complications, such as epilepsy-related injuries and sudden unexpected death, affecting the health and quality of life of patients [6–8]. Moreover, the occurrence of unexpected seizures in public settings frequently engenders a profound sense of stigma among individuals with epilepsy, significantly affecting their mental well-being and potentially leading to psychological disorders, including depression [9, 10]. Identifying biomarkers and early warning signs along with implementing timely interventions to mitigate seizure propensity may constitute an effective strategy for addressing these issues.

The pathophysiology of epilepsy remains complex and has not yet been fully elucidated. Elevated levels of proinflammatory cytokines have been observed in epileptogenic foci [11, 12]. Anti-inflammatory agents such as miconazole have been shown to reduce inflammatory mediators and decrease seizure frequency [13]. Furthermore, the interleukin-6 (IL-6) blocker tocilizumab has demonstrated efficacy in treating refractory status epilepticus [14]. These findings underscore the pivotal role of inflammation in ES pathophysiology and management. A range of inflammatory markers, such as body temperature, high-sensitivity C-reactive protein (CRP) [15], procalcitonin, IL-6, IL-1*β*, tumor necrosis factor-alpha (TNF-*α*), and high-mobility group box 1 (HMGB-1), have been extensively investigated as potential biomarkers for ES [16–20]. However, the current research has only established a significant association between these markers and ES. Epilepsy is one of the most prevalent neurological disorders worldwide, affecting over 70 million individuals [3]. Consequently, there is an urgent need to develop inexpensive and rapid biomarkers for diagnosis and management.

The neutrophil-to-lymphocyte ratio (NLR) is an accessible marker of systemic inflammation that is associated with various diseases, including cardiovascular and respiratory diseases and cancer [21–25], and that reflects an immune response balance and enhanced inflammatory response. Previous studies have found that an elevated NLR is often accompanied by ES, suggesting a possible connection between the NLR and ES [26, 27]. However, the link between the NLR and ES remains unclear, with studies showing mixed results [28]. This inconsistency may stem from differences in the study design and population. Further research is needed to clarify the role of the NLR as an ES biomarker.

In this study, we retrospectively analyzed 414 epilepsy cases to explore the association between NLR and ES. We compared NLR values in patients with epilepsy with and without ES at admission to explore the potential of NLR as an ES biomarker and investigate potential underlying factors contributing to the relationship.

## 2. Methods and Materials

### 2.1. Patients and Grouping

A total of 454 patients with epilepsy admitted to the Department of Emergency Neurology at Guizhou Medical University Affiliated Hospital between 2017 and 2024 were retrospectively analyzed. The inclusion criteria were as follows: (1) diagnosis based on the diagnostic criteria outlined by the International League Against Epilepsy (ILAE) guidelines and (2) hospitalization. The exclusion criteria were as follows: (1) incomplete data, (2) age < 18 years, and (3) comorbidities, including renal failure, respiratory failure, anemia, malignancies (e.g., undergoing radiotherapy or chemotherapy), stressful conditions (e.g., surgery and severe trauma), and endocrine disorders, including hyperthyroidism and hypothyroidism. Finally, 40 patients were excluded.

The participants were divided into two groups: an experimental group that included patients with ES and a control group that included patients with epilepsy who visited the hospital for other reasons.

### 2.2. Observation Indexes

The observational indices encompassed patient demographics including sex, age, body mass index (BMI), and vital signs at admission. In addition, medical data such as records of intracranial infections (e.g., encephalitis, meningitis, and brain abscess), traumatic brain injuries, intracranial tumors (primary or metastatic), and cerebrovascular diseases (e.g., cerebral hemorrhage, subarachnoid hemorrhage, cerebral infarction, cerebral aneurysm, and cerebral arteriovenous malformations) were considered. Additionally, the patients' initial hematological inflammation indices were collected.

### 2.3. Hematological Inflammation Indexes

We systematically collected data on the absolute neutrophil count (ANC), neutrophil percentage, lymphocyte percentage, absolute lymphocyte count (ALC), and white blood cell (WBC) count from the initial routine blood tests conducted postadmission for all included cases. Data underwent a thorough statistical analysis.

### 2.4. NLR

The NLR was quantified by calculating the ratio of neutrophils to lymphocytes. In a previous study [29], the NLR was calculated as the ratio of ANC to ALC. Notably, the percentage of neutrophils divided by the percentage of lymphocytes is mathematically equivalent to ANC/ALC. In our study, the percentages of neutrophils and lymphocytes exhibited a normal distribution. Consequently, the ratio of the percentage of neutrophils to that of lymphocytes was used to represent the NLR. We stratified the NLR according to the incidence of ES. We initially divided each NLR range (e.g., 1–2, 2–3, and 3–4) into separate tiers to ensure that the values did not exceed the upper limit of each range. To facilitate the statistical analysis, we merged tiers that exhibited similar seizure rates.

### 2.5. Sample Size Estimation

The sample size was determined based on a case-control study. The formula was *n* = ([*Z*_*α*_ + *Z*_*β*_] [2] × 2 × $\overset{―}{p}$ × [1−$\overset{―}{p}$])/(*P*_1_−*P*_0_) [2], where $\overset{―}{p}$ = (*P*_1_ + *P*_0_)/2 is the average exposure probability, *P*_1_ is the exposure probability in the experimental group, and *P*_0_ is the exposure probability in the control group. The parameter settings were determined based on the results of preliminary experiments: *Z_α_* = 1.96, *Z*_*β*_ = 1.282, *P*_0_ = 0.58, and *P*_1_ = 0.74. The calculated sample size was 111. To account for samples that did not meet the criteria, an additional 20% was added to the calculated sample size; thus, the minimum total required was 134.

### 2.6. Statistical Methods

SPSS (version 21.0) software was used for the statistical analyses. Quantitative data that conform to a normal distribution are reported as mean ± standard deviation (mean ± SD). *t*-Tests were used to compare two groups, whereas one-way ANOVA was used for multigroup comparisons. Quantitative data that did not follow a normal distribution are presented as medians and interquartile ranges (Med [Q1–Q3]). The Mann–Whitney *U* test was used to compare two groups, whereas the Kruskal–Wallis test was used for multigroup comparisons. Count data are expressed as *n* (%), and differences were tested using cross-tabulation. Statistical significance was set at *p*-values < 0.05.

## 3. Results

### 3.1. General Patient Data

Among the 454 included patients, 40 were excluded because 11 were younger than 18 years of age, 10 had incomplete data, and 19 had comorbidities. Ultimately, 414 patients were included in the analysis (284 males and 151 females, aged 18–90 years [mean: 48.4 ± 18.4 years]). The experimental group included 271 patients, and the control group included 143 patients. The mean body temperature was 36.6 (36.5–36.9)°C (range: 34.0°C–39.4°C). Compared with the control group, patients without seizures had a higher rate of brain tumors (25.5% vs. 35.7%; *p*=0.031) and cerebrovascular diseases (31.7% vs. 42.7%; *p*=0.031), while no significant differences were observed between the two groups in terms of sex, age, BMI, systolic blood pressure (SBP), diastolic blood pressure (DPB), pulse, hospitalization duration, or residence (all *p*  > 0.05) (Table 1). We further analyzed the factors that led to acute ES. The results demonstrated significantly higher body temperatures in the ES group (36.6 [36.5–37.0]) than in the control group (36.6 [36.5–36.8]) (*p*=0.012) (Table 1). Although hyperthermia was not observed in either group, the findings indicated a significant correlation between elevated body temperature and the incidence of ES. We further analyzed whether patients in both cohorts were exposed to infection during or shortly before hospital admission. The criteria for evaluating infection included an elevated body temperature, cold symptoms, or a confirmed diagnosis of infection at the time of admission. The incidences of infection in the ES and control groups were 52.0% and 37.8%, respectively (*p*=0.004). Pulmonary, urinary tract, and intracranial infections were also identified. The incidence of intracranial infection was 16.2%, with no significant difference between the two groups (17.3% vs. 14.0%; *p*=0.403). Similarly, the overall prevalence of urinary tract infections was 4.1%, with no statistically significant difference between the groups (4.8% vs. 2.8%; *p*=0.438). The patients enrolled in this study predominantly experienced pulmonary infections, with a high overall incidence of 37.4%. Specifically, the observation group exhibited a significantly higher incidence rate than the control group (41.0% vs. 30.8%; *p*=0.026) (Table 1).

Next, a binary logistic regression model was employed to investigate the association between the presence of risk factors and the outcome of interest. A stepwise (conditional) approach was applied, which allowed the selection of the best predictors for the model based on their statistical significance. The predictors included in the model were infections, pulmonary infections, brain tumors, cerebrovascular diseases, and body temperature. The model was adjusted for potential confounders, and the final equation incorporated infection-related variables. As shown in Table 2, patients with epilepsy with infections had nearly double the risk of ES compared to those without infections (OR = 1.788, 95% CI: 1.182–2.703).

### 3.2. Hematological Inflammation Indexes in Patients With and Without ES

The mean percentage of neutrophils was 70.10% ± 14.20% (range: 34.2%–97.2%). No statistically significant difference was observed between the groups (70.80% ± 14.70% vs. 68.70% ± 13.20%; *p*=0.159). The mean percentage of lymphocytes was 20.30% ± 11.70% (range: 1.4%–59.4%); similarly, no statistically significant difference was found between the groups (20.00% ± 12.30% vs. 20.70% ± 10.60%; *p*=0.528). No statistically significant differences in ANC, ALC, or WBC counts were found between the two groups (all *p*  > 0.05). Next, we analyzed the NLR. The NLR ranged from 0.59 to 67.57 (3.81 [2.16–7.12]), and no statistically significant differences were observed between the groups (3.99 [2.16–7.75] vs. 3.20 [2.17–5.56]; *p*=0.164) (Table 3).

### 3.3. Association Between NLR and ES

**Figure 1:**
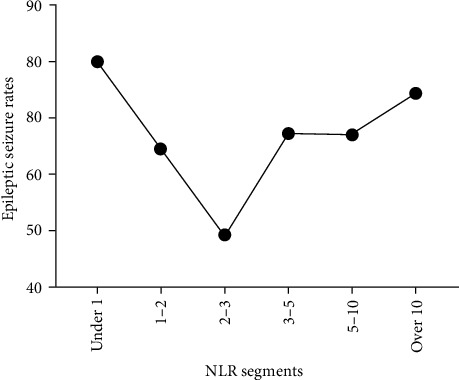
Epileptic seizure rates in different NLR tiers. NLR, neutrophil-to-lymphocyte ratio.

We observed that certain NLRs were correlated with comparable incidence of ES, specifically in the ranges of 3–4 and 4–5. Consequently, these ranges were combined into a unified tier. Using this iterative process, the data were classified into six distinct NLR tiers: <1, 1–2, 2–3, 3–5, 5–10, and >10. Unexpectedly, the analysis, revealed a “V”-shaped relationship between the NLR and ES (Figure 1). The incidence of ES was notably higher in individuals with NLRs < 2 (64.5%–80.0%) and >3 (67.3%–74.4%). The lowest ES incidence (49.2%) occurred in the NLR range of 2–3, at the lowest point of the “V” (Table 4). A comparative analysis of ES rates across various NLR tiers demonstrated that the ES rate at the nadir of the “V” was significantly lower than that observed in other tiers (*p*  < 0.05). No statistically significant differences in seizure rates were identified between the 3–5, 5–10, and >10 NLR tiers (*p*  > 0.05). The results are shown on the right side of Table 4.

### 3.4. Neutrophil and Lymphocyte Levels in the NLR Tiers

**Figure 2:**
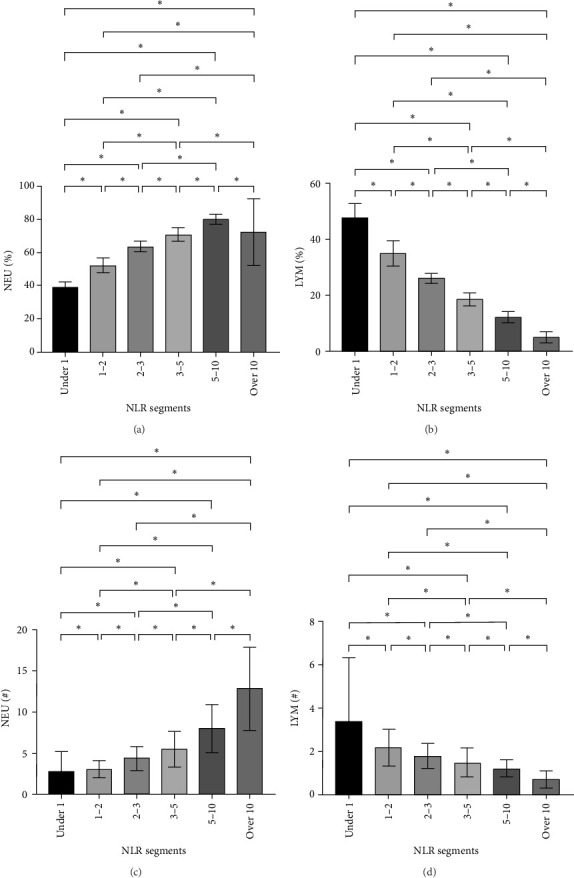
(A–D) Neutrophil and lymphocyte comparisons across groups in various NLR tiers. *⁣*^*∗*^*p*  < 0.05. NLR, neutrophil-to-lymphocyte ratio.

After NLR segmentation, the levels of both neutrophils and lymphocytes exhibited a distinct and approximately linear trend of variation rather than a “V”-shaped relationship. The percentage of neutrophils and ANC was positively correlated with an increase in the NLR tier, whereas the percentage of lymphocytes and ALC demonstrated the opposite trend. The neutrophil percentage in cases in the NLR tier of >10 was more than twice that in cases in the NLR tier of <1, and the ANC was more than four times higher. Conversely, in cases in the NLR tier of <1, the lymphocyte percentage was more than eight times higher and the ALC was nearly five times greater than that in cases in the NLR tier of >10 (Table 5). Statistically significant differences were observed between the tiers (*p*  < 0.05) (Figure 2). For populations with an NLR of 5–10 and >10, the mean neutrophil percentage was higher than normal, whereas for an NLR of <1, it was lower than the normal range of 40%–75%. The average ANC for cases in the NLR tier of >5 exceeded the normal 1.8–6.3 × 10^9^/L range. The lymphocyte percentage for cases in NLR tiers >3–5 was lower than the normal range of 20%–50%. Cases in the NLR tiers of >10 and <1 had abnormal ALCs outside the normal range of 1.1–3.2 × 10^9^/L. Neutrophil and lymphocyte abnormalities were observed in cases in both tier extremities. In particular, cases in the NLR tiers of 5–10 and higher had notably increased absolute counts and percentage of neutrophils, which was accompanied by a significant decrease in the absolute counts and percentage of lymphocytes. In patients with an NLR value <1, the percentage of neutrophils was abnormally reduced.

### 3.5. Other Factors Within the NLR Tiers

**Figure 3:**
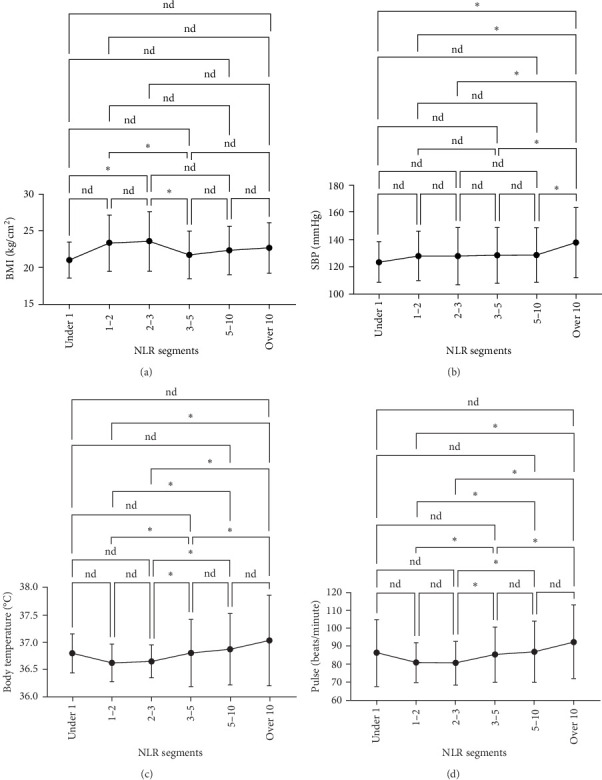
Factor comparisons across groups with different numbers of NLR tiers: (A) BMI; (B) systolic blood pressure; (C) body temperature; and (D) pulse comparison. *⁣*^*∗*^*p* < 0.05. BMI, body mass index; NLR, neutrophil-to-lymphocyte ratio.

**Figure 4:**
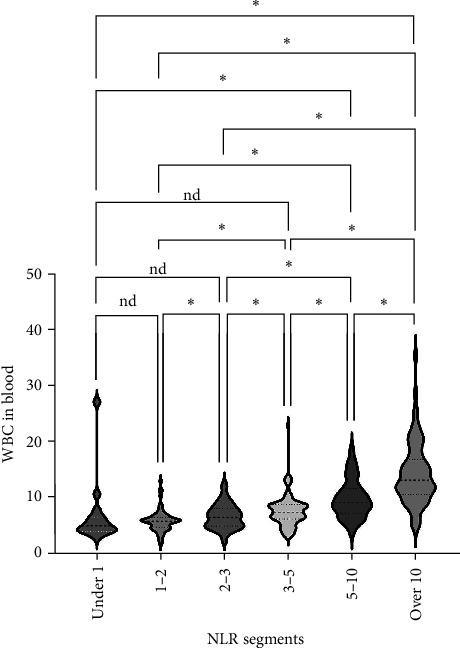
White blood cell (WBC) comparisons across groups in various NLR tiers. *⁣*^*∗*^*p* < 0.05. NLR, neutrophil-to-lymphocyte ratio.

We analyzed the demographic characteristics, vital signs, medical histories, and presence of infection in patients with different NLR tiers (Table 5). No statistically significant differences in sex, residence, DBP, length of hospital stay, or craniocerebral trauma were identified (all *p*  > 0.05). Patients with an NLR < 1 were the youngest (38.8 ± 17.1 years) compared to those with an NLR of 2–3 (50.3 ± 16.9), 5–10 (51.4 ± 19.9), and >10 (51.0 ± 18.9), with a significant age difference (*p*=0.029) (Table 5). The lowest MBI scores were observed in patients with an NLR <1 (21.4 ± 2.5) and those with NLRs of 3–5 (21.8 ± 3.5), while the highest was observed in patients with NLRs of 1–2 (23.2 ± 3.6) and 2–3 (23.7 ± 4.0) (Figure 3A). In addition, patients with NLRs of 1–2 (36.6 [36.5–36.6]) and 2–3 (36.6 [36.5–36.7]) had the lowest body temperatures; patients with NLRs of 3–5 (36.6 [36.5–36.9]) and higher (36.8 [36.5–37.4]) had a progressively increasing trend (Figure 3C). The results for pulse were similar to those for body temperature (Figure 3D). Patients in the NLR tier of >10 had significantly higher SBP than those in the other tiers (*p*=0.013) (Figure 3B). Patients in NLR tiers 2–3 (42.3%) and 3–5 (30.7%) were more likely to have craniocerebral tumors, while patients in tiers 1–2 (53.9%) were more frequently exposed to cerebrovascular diseases (*p*=0.008). The most compelling finding was that patients in the NLR tier of <2 exhibited the lowest infection rates (~25.0%), whereas those in the NLR tier of >5 demonstrated the highest infection rates (~60.0%). Patients in the NLR tier of 2–5 had an intermediate infection rate of ~40.0%. WBC counts were the lowest in patients in the NLR tier of 1–2, similar to those in the NLR tier of <1. For patients in the NLR tiers of 2–3 and higher, WBC counts increased by ~1 × 10^9^/L per tier. Once the NLR exceeded 10, WBC counts increased sharply to >14 × 10^9^/L, well beyond the normal range (3.5–9.5 × 10^9^/L) (Figure 4). In summary, high NLRs result from unusually increased levels of neutrophils and low levels of lymphocytes associated with infections, whereas low NLRs are due to relatively fewer neutrophils and more lymphocytes, possibly due to other factors.

## 4. Discussion

Neutrophils and lymphocytes are the two major cell types in the immune system. Neutrophils reflect the persistence of inflammation, whereas lymphocytes represent the immune regulatory pathways. The NLR is a simple, cost-effective, and stable indicator that provides comprehensive feedback on inflammation and immune regulation. It is increasingly used as a biomarker for disease severity and prognosis in inflammatory conditions [30, 31]. Previous research on the association between an elevated NLR and ES has been inconclusive. Some studies indicate a positive correlation and suggest that an elevated NLR is a strong predictor of ES [26, 32–35], whereas others have found no significant relationship [36]. Our study provides novel insights into the relationship between the NLR and ES. Contrary to the expected straightforward association, we observed a “V”-shaped correlation between NLR tiers and the incidence of ES. This finding suggests that both very low and very high NLRs are associated with an increased risk of seizures, indicating a complex interplay between the NLR and seizure susceptibility.

In our study, the lowest incidence of seizures (49.2%) was observed at an NLR of 2–3. Importantly, in this cohort, 42.3% of the patients had a history of brain tumors and 40.8% had been exposed to infections. Studies indicate that brain tumors significantly contribute to focal seizures, affecting 40%–70% of patients with a high recurrence rate [37–39]. Our findings are consistent with those of previous studies, highlighting the role of structural abnormalities in seizures. Notably, an NLR of 2–3 may represent a critical threshold associated with a decreased likelihood of seizure occurrence in individuals with epilepsy. A comprehensive study involving a large pan-cancer cohort demonstrated that patients with an NLR of 2–3 exhibited the most favorable treatment outcomes, characterized by improved survival rates and enhanced treatment responses. This observation suggests that an NLR within this range may reflect an optimal balance between the innate (neutrophils) and adaptive (lymphocytes) immune responses [25]. Consequently, we propose that an NLR of 2–3 may also serve as an equilibrium point that influences seizure activity in patients with epilepsy.

Neutrophils play a key role in regulating neuronal hyperexcitability and can trigger seizures by infiltrating the brain [40]. However, their volatility and lack of accuracy render them unsuitable biomarkers. Studies have suggested that a higher NLR reflects a strong inflammatory response and imbalance in the immune system, making it a more suitable biomarker [41–43]. The inflammatory mechanisms underlying epilepsy have attracted considerable scholarly interest, particularly concerning their association with an altered NLR and ES. An elevated NLR is an independent predictor of recurrent seizures, suggesting that the NLR could serve as a biomarker of seizure severity and recurrence [32]. However, the precise nature of the relationship between variations in the NLR and ES remains unclear. Despa, Musteata, and Solcan [44] found NLRs of 6.49 for idiopathic epilepsy, 8.19 for structural epilepsy, and 3.84 in healthy dogs. Ebner et al. [35] identified an NLR of 2.98 as the optimal cutoff for predicting poststroke seizures. Li et al. [28] reported a median NLR of 4.26 for autoimmune encephalitis with seizures and 2.62 without. Huang et al. [33] found a mean NLR of 4.5 for generalized tonic–clonic seizures and 2.6 for the normal group, aligning with the findings of Li et al. [28]. We found that an NLR > 3 was positively correlated with an elevated risk of ES, with age and active infection exerting a significant influence on this association. Previous research has suggested that an NLR > 3 may indicate immune dysregulation [25]. Consequently, an NLR > 3 signifies a heightened inflammatory response and immune dysregulation, which can serve as a critical threshold for predicting the occurrence of ES.

Our investigation revealed that an NLR of <2 is significantly correlated with an increased incidence of ES. In this cohort, characterized by a relative decrease in neutrophils and an increase in lymphocytes without notable alterations in the overall WBC count, the association between the NLR and ES does not appear to be attributable to inflammatory responses. One study indicated that an NLR of <2 may represent another type of immune dysregulation [25]. Importantly, this group exhibited a marked increase in the incidence of cerebrovascular disease. Cerebrovascular diseases can lead to ES via complex mechanisms. This process involves excitotoxicity, inflammation, and changes in the blood–brain barrier, all of which increase neuronal hyperexcitability and the risk of seizures [45]. Reports indicate that patients experiencing seizures within 1 week of a stroke have a 33% recurrence risk, which increases to 75% if seizures occur after 1 week [46]. We believe that an NLR of <2 strongly predicts ES, which is often linked to immune dysregulation and cerebrovascular disease exposure.

When interpreting our results, several limitations must be noted, including the small sample size and absence of healthy controls; the observational nature of the study, which prevents establishing causality between the NLR and ES incidence; the lack of a temporal relationship between NLR changes and seizure onset; and potentially unaccounted confounding factors, such as medications, comorbidities, and lifestyle. Future research should address these issues to gain a clearer understanding of the role of the NLR in ES.

## 5. Conclusion

The NLR is associated with ES and could be a reliable predictive biomarker. However, the correlation between the NLR and ES does not follow a linear pattern and instead exhibits a “V”-shaped trajectory. Both arms of the “V” are associated with an increased incidence of ES. To the left of the “V,” the NLR may be associated with the patient's medical history, including cerebrovascular diseases and immune dysregulation; to the right of the “V,” the NLR may be correlated with the severity of the patient's condition and a pronounced inflammatory response. An NLR > 3 could predict seizures in inflammatory conditions, whereas an NLR < 2 might indicate seizures in noninflammatory conditions.

## Figures and Tables

**Table 1:** General data of cases with/without epileptic seizure.

Variables	Total	Cases with epileptic seizure	Cases without epileptic seizure	*p*-Value/*Z*-value/*χ*^2^-value
	*n* = 414	*n* = 271	*n* = 143	
Age (years), mean ± SD	48.4 ± 18.4	48.4 ± 18.9	48.3 ± 17.4	0.981^g^
Gender, *n* (%)				
Male	268 (64.7)	179 (66.1)	89 (62.2)	0.451^f^
Female	146 (35.3)	92 (33.9)	54 (37.8)	
BMI (kg/m^2^), mean ± SD	22.6 ± 3.6	22.7 ± 3.6	22.2 ± 3.3	0.229^g^
*T* (°C), med (Q1–Q3)	36.6 (36.5–36.9)	36.6 (36.5–37.0)	36.6 (36.5–36.8)	0.012^g^
SBP (mmHg), mean ± SD	129.8 ± 21.4	130.4 ± 21.0	128.7 ± 22.2	0.448^g^
DBP (mmHg), mean ± SD	80.9 ± 14.3	80.8 ± 14.2	81.1 ± 14.4	0.865^g^
Hospitalization duration (days), mean ± SD	11.9 ± 8.7	11.4 ± 8.4	13.0 ± 12.1	0.146^g^
*P* (count/minute), med (Q1–Q3)	82 (75–92)	82 (74–94)	81.0 (76–91)	0.859^e^
Residence, *n* (%)				
Citiy	157 (37.9)	106 (39.1)	51 (35.7)	0.594^f^
Town or countryside	257 (62.1)	164 (60.5)	92 (64.3)	
Craniocerebral trauma^a^, *n* (%)				
With	49 (11.8)	32 (11.8)	17 (11.9)	1.000^f^
Without	365 (88.2)	239 (88.2)	126 (88.1)	
Brain tumor^b^, *n* (%)				
With	120 (29.0)	69 (25.5)	51 (35.7)	0.031^f^
Without	294 (71.0)	202 (74.5)	92 (63.3)	
CVD^c^, *n* (%)				
With	147 (35.5)	86 (31.7)	61 (42.7)	0.031^f^
Without	267 (64.5)	185 (68.3)	82 (57.3)	
Infection^d^, *n* (%)				
With	195 (47.1)	141 (52.0)	54 (37.8)	0.004^f^
Without	219 (52.9)	130 (48.0)	89 (62.2)	
Respiratory infection				
With	155 (37.4)	111 (41.0)	44 (30.8)	0.026^f^
Without	259 (62.6)	160 (59.0)	99 (69.2)	
Urinary tract infections, *n* (%)				
With	17 (4.1)	13 (4.8)	4 (2.8)	0.438^f^
Without	397 (95.9)	258 (95.2)	139 (97.2)	
Intracranial infections, *n* (%)				
With	67 (16.2)	47 (17.3)	20 (14.0)	0.403^f^
Without	347 (83.8)	224 (82.7)	123 (86.0)	

*Note: p*-Value < 0.05, *Z*-value < 0.05, *χ*^2^-value < 0.05 indicated that the difference was statistically significant. *T*, body temperature.

Abbreviations: BMI, body mass index; CVD, cerebrovascular diseases; DBP, diastolic blood pressure; *P*, pulse; SBP, systolic blood pressure.

^a^Represented by traumatic brain injury.

^b^Including primary tumor (*n* = 114) and metastatic tumor (*n* = 6).

^c^Including cerebral hemorrhage (*n* = 24), subarachnoid hemorrhage (*n* = 14), cerebral infarction (*n* = 6), cerebral aneurysm (*n* = 68), and cerebral arteriovenous malformations (*n* = 35).

^d^Including respiratory infection (*n* = 155), urinarytract infection (*n* = 17), or intracranial infection (*n* = 67).

^e^“Mann–Whitney U” test was used for statistical analysis.

^f^“Cross-tabulations” was used for statistical analysis.

^g^“*t*-Test” was used for statistical analysis.

**Table 2:** Binary logistic regression analysis of factors influencing epileptic seizures.

Variables	Estimate (*B*)	Standard error (SE)	Wald *χ*²	*p*-Value	Exp (*B*)	95% CI
Infection	0.581	0.211	7.576	0.006	1.788	1.182–2.703

*Note:* Encoding method, epileptic seizures: 1 (with), 0 (without); infection: 1 (with), 0 (without); respiratory infection: 1 (with), 0 (without); brain tumor:1 (with), 0 (without); and cerebrovascular diseases: 1 (with), 0 (without). Body temperature, respiratory infection, brain tumor, cerebrovascular diseases, and infection were included in the binary regression model. Method, forward stepwise (conditional). Steps 1a, infection.

**Table 3:** The hematological inflammation indexes on cases with/without epileptic seizure.

Variables	Total	Cases with epileptic seizure	Cases without epileptic seizure	*p*-Value/*Z*-value
	414	271	143	—
WBC (10^9^/L), med (Q1–Q3)	7.85 (5.95–10.67)	8.06 (5.97–10.79)	7.24 (5.82–10.09)	0.270^b^
NEU (%), mean ± SD	70.10 ± 14.20	70.80 ± 14.70	68.70 ± 13.20	0.159^a^
LYM (%), mean ± SD	20.30 ± 11.70	20.00 ± 12.30	20.70 ± 10.60	0.528^a^
NLR, med (Q1–Q3)	3.81 (2.16–7.12)	3.99 (2.16–7.75)	3.20 (2.17–5.56)	0.164^b^
ANC (10^9^/L), med (Q1–Q3)	5.31 (3.46–8.1)	5.60 (3.62–8.50)	4.88 (3.34–7.20)	0.191^b^
ALC (10^9^/L), med (Q1–Q3)	1.37 (0.92–1.93)	1.36 (0.83–1.90)	1.37 (1.04–1.99)	0.411^b^

*Note:* “%” denoted percentage; *p*-value < 0.05, *Z*-value < 0.05 indicated that the difference was statistically significant.

Abbreviations: ALC, absolute lymphocyte count; ANC, absolute neutrophil count; LYM, lymphocyte; NEU, neutrophil; WBC, white blood cell.

^a^“*t*-Test” was used for statistical analysis.

^b^“Mann-Whitney U” test was used for statistical analysis.

**Table 4:** Epileptic seizure rate in different NLR segments.

Epileptic seizure				NLR segments					
NLR segments	Total	With (*n* = 271)	Without (*n* = 143)	Under 1	1–2	2–3	3–5	5–10	Over 10
	414	271	143	—	—	—	—	—	—
Under 1	15 (3.6)	12 (80.0)	3 (20.0)	—	*p*=0.018	*p* < 0.001	*p*=0.054	*p*=0.054	*p*=0.401
1–2	76 (18.4)	49 (64.5)	27 (35.5)	*p*=0.018	—	*p*=0.033	*p*=0.767	*p*=0.767	*p*=0.170
2–3	71(17.1)	35 (49.2)	36 (50.7)	*p* < 0.001	*p*=0.033	—	*p*=0.015	*p*=0.015	*p* < 0.001
3–5	101 (24.4)	68 (67.3)	33 (32.7)	*p*=0.054	*p*=0.767	*p*=0.015	—	*p*=1.000	*p*=0.352
5–10	73 (17.6)	49 (67.1)	24 (32.9)	*p*=0.054	*p*=0.767	*p*=0.015	*p*=1.000	—	*p*=0.352
Over 10	78 (18.8)	58 (74.4)	20 (25.6)	*p*=0.401	*p*=0.170	*p* < 0.001	*p*=0.352	*p*=0.352	—

*Note:* The table on the left delineates the seizure rates across various NLR value ranges, whereas the table on the right illustrates the intergroup comparison of seizure rates within these specified NLR ranges. The findings revealed that the seizure rate is lowest in the cohort with NLR values between 2 and 3, with this difference being statistically significant compared to other groups (*p* < 0.05).

Abbreviation: NLR, neutrophil-to-lymphocyte ratio.

**Table 5:** Detailed information on cases with different NLR segments.

Variables	Total	Under 1	1–2	2–3	3–5	5–10	Over 10	*p*-Value
	414	15	76	71	101	73	78	
Age (years), mean ± SD	48.4 ± 18.4	38.8 ± 17.1	44.2 ± 16.7	50.3 ± 16.9	47.6 ± 18.4	51.4 ± 19.9	51.0 ± 18.9	0.029^a^
Gender, *n* (%)								
Male	268 (64.7)	11(73.3)	49(64.5)	39(54.9)	64(63.4)	47 (64.4)	58 (74.4)	0.242^c^
Female	146 (35.5)	4(26.7)	27(35.5)	32(45.1)	37(36.6)	26 (35.6)	20 (25.6)	
BMI (kg/m^2^), mean ± SD	22.6 ± 3.6	21.4 ± 2.5	23.2 ± 3.6	23.7 ± 4.0	21.8 ± 3.5	22.1 ± 3.1	22.6 ± 3.4	0.006^a^
*T* (°C), med (Q1–Q3)	36.6 (36.5–36.9)	36.6 (36.5–36.9)	36.6 (36.5–36.6)	36.6 (36.5–36.7)	36.6 (36.5–36.9)	36.6 (36.5–37.1)	36.8 (36.5–37.4)	<0.001^b^
SBP (mmHg), mean ± SD	129.8 ± 21.4	123.5 ± 15.1	127.8 ± 18.4	127.8 ± 21.1	128.3 ± 20.5	128.5 ± 20.1	137.8 ± 26.0	0.013^a^
DBP (mmHg), mean ± SD	80.0 (72.0–89.0)	78.0 (72.0–91.0)	80.0 (74.0–87.0)	80.0 (73.0–88.0)	78.0 (70.0–91.0)	80.0 (67.0–86.0)	81.0 (71.8–93.3)	0.633^b^
Hospitalization duration (days), med (Q1–Q3)	10.0 (5.0–17.0)	9.0 (7.0–14.0)	7.0 (4.0–19.0)	12.0 (4.0–18.0)	10.0 (5.0–17.0)	11(6.0–17.8)	9(4.0–18.0)	0.605^b^
*P* (beats/min), med (Q1–Q3)	85.4 ± 16.1	86.3 ± 18.4	81.0 ± 11.1	80.7 ± 12.1	85.4 ± 15.4	87.0 ± 16.9	92.4 ± 20.4	<0.001^a^
Residence, *n* (%)								
Citiy	157 (37.9)	9 (60.0)	31 (40.8)	24 (33.8)	33 (32.7)	32 (43.8)	28 (35.9)	0.279^c^
Town or countryside	257 (62.1)	6 (40.0)	45 (59.2)	47 (66.2)	68 (67.3)	41 (56.5)	50 (64.1)	—
Craniocerebral trauma, *n* (%)								
With	49 (11.8)	3 (20.0)	7 (9.2)	7 (9.9)	14 (13.9)	8 (11.0)	10 (12.8)	0.814^c^
Without	365 (88.2)	12 (80.0)	69 (90.8)	64 (90.1)	87 (86.1)	65 (89.0)	68 (87.2)	—
Brain tumor, *n* (%)								
With	120 (29.0)	4 (26.7)	23 (18.4)	30 (42.3)	31 (30.7)	13 (17.8)	19 (24.2)	0.041^c^
Without	294 (71.0)	11 (73.3)	53 (69.7)	41 (57.7)	70 (69.3)	60 (82.2)	59 (75.6)	—
CVD, *n* (%)								
With	147 (35.5)	4 (26.7)	41 (53.9)	21 (29.6)	28 (27.7)	26 (35.6)	27 (34.6)	0.008^c^
Without	267 (64.5)	11 (73.3)	35 (46.1)	50 (70.4)	73 (72.3)	47 (64.4)	51 (65.4)	—
Infection, *n* (%)								
With	195 (47.1)	4 (26.7)	19 (25.0)	29 (40.8)	47 (46.5)	44 (60.3)	52 (66.7)	<0.001^c^
Without	219 (52.9)	11 (73.3)	57 (75.0)	42 (59.2)	54 (53.5)	29 (39.7)	26 (33.3)	—
WBC (10^9^/L), mean ± SD	8.9 ± 4.6	7.0 ± 5.9	6.0 ± 1.9	6.9 ± 2.2	7.8 ± 2.8	10.0 ± 3.4	14.4 ± 5.5	<0.001^a^
NEU (%), mean ± SD	70.1 ± 14.2	39.1 ± 2.9	52.2 ± 4.6	63.5 ± 3.3	70.8 ± 4.1	80.1 ± 3.1	89.1 ± 4.0	<0.001^a^
LYM (%), mean ± SD	20.3 ± 11.7	47.8 ± 5.1	35.0 ± 4.5	26.1 ± 1.9	18.6 ± 2.3	12.2 ± 2.0	5.1 ± 2.0	<0.001^a^
ANC (10^9^/L), mean ± SD	12.8 ± 5.1	2.8 ± 2.5	3.1 ± 1.0	4.4 ± 1.5	5.5 ± 2.1	8.0 ± 2.8	12.9 ± 5.1	<0.001^a^
ALC (10^9^/L), med (Q1–Q3)	1.37 (0.92–1.93)	2.46 (2.01–3.38)	2.10 (1.70–2.35)	1.74 (1.33–2.13)	1.37 (1.17–1.65)	1.12 (0.89–1.47)	0.63 (0.46–0.90)	<0.001^b^

*Note:* “%” denoted percentage; *p*-value < 0.05, Z-value < 0.05, *χ*^2^-value < 0.05 indicated that the difference was statistically significant. *T*, body temperature.

Abbreviations: ALC, absolute lymphocyte count; ANC, absolute neutrophil count; BMI, body mass index; CVD, cerebrovascular diseases; DBP, diastolic blood pressure; LYM, lymphocyte; NEU, neutrophil; NLR, neutrophil-to-lymphocyte ratio; *P*, pulse; SBP, systolic blood pressure; WBC, white blood cell.

^a^“*t*-Test” was used for statistical analysis.

^b^“Mann–Whitney U” test was used for statistical analysis.

^c^“Cross-tabulations” was used for statistical analysis.

## Data Availability

All data generated or analyzed during this study are included in this article.
